# p16 as a diagnostic marker of cervical neoplasia: a tissue microarray study of 796 archival specimens

**DOI:** 10.1186/1746-1596-4-22

**Published:** 2009-07-09

**Authors:** Iana Lesnikova, Marianne Lidang, Stephen Hamilton-Dutoit, Jørn Koch

**Affiliations:** 1 Institute of Pathology, Aarhus University Hospital, Aarhus, Denmark; 2 Institute of Pathology, Herlev Hospital, Herlev, Denmark

## Abstract

**Background:**

To evaluate the usefulness of this biomarker in the diagnosis of cases of cervical neoplasia we studied the immunohistochemical expression of p16^INK4a ^in a large series of archival cervical biopsies arranged into tissue microarray format.

**Methods:**

TMAs were constructed with tissue cores from archival formalin fixed, paraffin-embedded donor tissues from 796 patients, and included cases of cervical intraepithelial neoplasia (CIN)1 (n = 249), CIN2 (n = 233), CIN3 (n = 181), and invasive cervical carcinoma (n = 133). p16^INK4a ^expression was scored using two different protocols: 1) positive *vs *negative p16^INK4a ^staining; 2) a semi-quantitative immunohistochemical score (0 to 8 points) according to the intensity of staining and the proportion of stained cells

**Results:**

p16^INK4A ^expression was not seen in normal cervix tissue, but was found with increasing frequency in the sequence: CIN1 (180/249; 72.3%) – CIN2 (212/233; 91.0%) – CIN3 (178/181; 98.3%) – invasive carcinoma (131/133; 98.5%). Using semi-quantitative scoring, all normal cervical samples had low scores (from 0 to 2 points), whilst the number of specimens with high scores was proportional to the degree of cervical dysplasia or the presence of invasive carcinoma.

**Conclusion:**

Immunohistochemical analysis of p16^INK4a ^expression is a useful diagnostic tool. Expression is related to the degree of histological dysplasia, suggesting that it may have prognostic and predicative value in the management of cervical neoplasia.

## Background

Human papillomavirus (HPV) infection is a critical factor in nearly all cases of cervical cancer [1-3]. Approximately 200 different subtypes of HPV have now been characterized, some of which carries a higher risk of cancer development than others. The great majority of human cervical cancers are associated with high-risk HPV infections, although such infections result in progression to cervical cancer in only a small percentage of infected women, and typically only after a long latency period [4].

The intracellular targets for HPVs include a number of regulatory proteins such as cyclins, cyclin dependent kinases, cyclin inhibitors, and cell cycle-associated proteins. The HPV E6 and E7 oncoproteins inactivate the p53 and retinoblastoma protein (RB) tumor suppressors, respectively, leading to hyperproliferation and genomic instability [4-7]. RB inhibits the progression of cells into S phase and is regulated by cyclin D1 via phosphorylation. Progressive and prolonged phosphorylation of the RB protein leads to its inactivation and to a reduction of its growth suppressive activity [7,8]. This inactivation is mediated by the release of E2F-like transcription factors from RB, which allows the activation of CDK and transcriptional activation of target promoters [9]. The CDKN2A gene product, the p16^INK4A ^protein, is a tumour suppressor protein that inhibits CDK4 and CDK6, which phosphorylate the RB protein. A reciprocal relationship between p16^INK4A ^and RB expression has been observed [10]. The p16^INK4A ^gene was found inactivated in a large percentage of tumor cell lines, suggesting that it was indeed a tumor suppressor gene [11-13].

p16^INK4A ^overexpression has been demonstrated in cervical cancers as a result of functional inactivation of RB by the HPV E7 protein [14]. It has been reported that the HPV negative cell line C33A and HPV negative adenocarcinomas are p16^INK4A ^positive, which indicates that a non-HPV dependent p16^INK4A ^expression pathway may also exist [15,16].

A number of studies have demonstrated that p16^INK4A ^may be a useful marker for squamous and glandular epithelial dysplasia in the uterine cervix [17,18]. Furthermore, expression of p16^INK4A ^appears to correlate with the degree of cervical neoplasia [19,20]. It was also recently reported that p16^INK4A ^immunostaining can be used for discriminating integrated from non-integrated HPV infections [18,21].

Tissue microarray (TMA) is a well-established technology for performing high-throughput gene expression analysis in tissue sections [22]. In this technique, small cores of formalin fixed paraffin embedded (FFPE) tissue are first removed from a large number of "donor" paraffin blocks, and then arrayed in a new "recipient" TMA. TMAs can contain samples from hundreds of different donor specimens, all of which can be stained simultaneously for a particular marker in a single experiment using immunohistochemistry or in situ hybridization. In the previously published TMA study of the uterine cervix, analysis of two tissue cores from cases of cervical adenocarcinomas and their pre-invasive precursors lesions in more than 95% of cases gave data comparable with that obtained from staining a whole tissue section [23,24].

The present study was conducted in order to study the immunohistochemical expression of p16^INK4A ^in a large number of archival sections of different degrees of precancerous lesions and cervical cancer using tissue microarray (TMA) technology and to find the optimal evaluation method of p16^INK4A ^expression for practical diagnostic purposes.

## Methods

### Tissue processing and TMA preparation

Study cases were randomly selected from the archive of the Institute of Pathology, Aarhus University Hospital. All cases were routine diagnostic surgical specimens including biopsies, loop, and cone excisions of the uterine cervix, and hysterectomy sections. Tissues were fixed in buffered formalin, processed using standard procedures and embedded in paraffin. Tissue blocks were stored at room temperature in the pathology archive up to 10 years before being used for TMA construction. All specimens were diagnosed by an experienced gynaecological pathologist according to World Health Organization classification criteria. Only the samples that completely fulfilled established diagnostic criteria were included in the study. Tissue blocks containing only small or otherwise inadequate samples were excluded. In all, 796 specimens were included in the study, comprising 249 cases of CIN1, 233 cases of CIN2, 181 cases of CIN3, and 133 cases of invasive cervical carcinoma. The last group included 105 squamous cell carcinomas (79%) and 28 adenocarcinomas (21%) to represent the incidence of various degree of dysplasia and cancer in whole population. 10 samples of normal uterine cervix were used as a control.

Histological slides for each specimen included in the TMA were reviewed, representative "donor" regions were identified under the microscope, and then these were marked up with ink on each glass slide. Pilot TMAs were constructed in order to identify the optimal core diameter of samples to be used. Cores with diameters of 0.6 mm, 1 mm, and 2 mm were compared with respect to ease of TMS construction, ease of sectioning, and representativity (data not shown). Cores of 1 mm diameter were chosen for the project TMAs, as these proved easy to handle and gave representative results compared with whole tissue sections, TMAs were constructed using a manual Tissue Arrayer (Beecher Instruments, Silver Spring, MD, USA) essentially as described [22]. From the previously defined area in the donor block, a 1 mm tissue cylinder was punched out and transferred into the recipient block. In some cases where the donor tissues were very thin, more than one tissue cylinder was stacked in the same hole in the recipient block. The number of cores used per case varied from one to ten, depending upon the size of the region of interest in each section. In the recipient block, cores were arrayed according to a defined x-y coordinate position. Normal liver and placenta tissue cores were used as position markers at one corner of each TMA. After construction, the TMA blocks were heated in an oven at 37°C for 30 min and then at 60°C for 10 min. All blocks were cut on a standard microtome. The 3 μm tissue sections were floated in a 45°C water bath and collected on Superfrost^® ^plus coated glass slides (Menzel-Gläser Gerhard Menzel, Glasbearbeitungswerk GmbH & Co. KG – Saarbrückener Str. 248 – D-38116 Braunschweig). Section cutting proved to be a critical step in TMA production, requiring considerable skill on the part of the microtome operator in order to section successfully all the tissue specimens in a TMA. Approximately 40 to 150 representative sections could be cut from a TMA block depending upon its size, the thickness of the donor tissues, and the experience of the operator.

### Immunostaining for p16^INK4A^

p16^INK4A ^was detected by immunohistochemistry using monoclonal murine antibody clone JC8 (Biocompare Inc., South San Francisco, CA 94080) on a BenchMark Autostainer (Ventana Medical Systems, Illkirch, France). Ready-to-use primary antibody optimally diluted according to the manufacturer's recommendations was purchased from Ventana Medical Systems. p16^INK4A ^expression was associated with distinct nuclear and cytoplasmic staining of epithelial cells and was evaluated using two different scoring protocols: 1) Positive (moderate or strong staining in more than 10% of epithelial cells) *vs *negative (less than 10% of epithelial cells with moderate or strong staining). 2) A semi-quantitative immunohistochemical score (0 to 8 points) depending on the intensity (0 – 3 points; 0 – no staining; 1 – weak staining; 2 points – moderate staining; and 3 – strong staining) and the proportion (0 – 5 points; 0 – no staining; 1 – <1% positive; 2 – 1% – 10% positive; 3 – 11% – 33% positive; 4 – 34% – 66% positive; and 5 – > 66% of positive) of stained cells [25].

Non-parametric statistic tests for trend across ordered groups were used.

## Results

**Figure 1:**
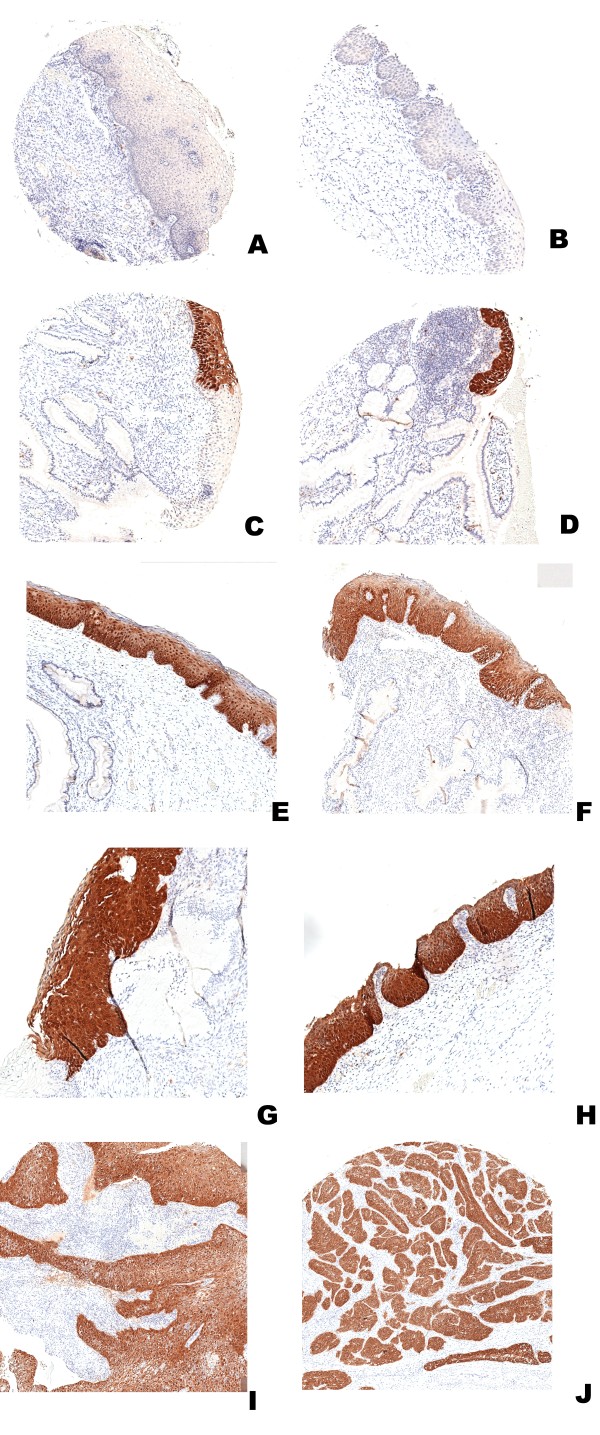
**Immunohistochemical analysis of p16^INK4A ^expression using monoclonal antibody clone JC8 in tissue microarray cores from cervix biopsies with normal epithelium (A, B), CIN1 (C, D), CIN2 (E, F), CIN3 (G, H) and invasive squamous carcinoma (I, J)**. The normal epithelial and stromal cells are negative (A – B). Strong, distinct, diffuse staining of both nuclei and cytoplasm is seen in the dysplastic/neoplastic epithelium (C – J).

p16^INK4A ^expression in epithelium was characterised by variable, weak to strong, diffuse nuclear and cytoplasmic staining. There was no difference in the intensity of staining between different epithelial layers. Clear and distinctive positive staining was observed only in dysplastic cells. Normal stromal and normal squamous epithelial cells were consistently negative (Figure 1). In some normal glandular epithelial cells diffuse weak or moderate staining was observed. We were not able to identify consistent staining patterns that we believed could discriminate between presumed integrated and non-integrated HPV.

### Evaluation of p16^INK4A ^immunostaining (see Figures 1 and Tables 1 and 2)

**Table 1:** Immunohistochemical analysis of p16^INK4A ^expression in tissue microarray cores from 806 cervix biopsies containing normal tissues, CIN1, CIN2, CIN3, and invasive cervical carcinoma (ICC), evaluated using the simple protocol.

		p16^INK4A ^immunostaining	
		
	n	Negative*	Positive^#^
Normal cervix	10	10 (100.0)	0 (0)
CIN1	249	69 (27.7)	180 (72.3)
CIN2	233	21 (9.0)	212 (91.0)
CIN3	181	3 (1.7)	178 (98.3)
ICC	133	2 (1.5)	131 (98.5)

*Negative: no staining or weak staining in less than 10% of epithelial cells.

^#^Positive: moderate or strong staining in more than 10% of epithelial cells.

**Table 2:** Immunohistochemical analysis of p16^INK4A ^expression in tissue microarray cores from 806 cervix biopsies containing normal tissues, CIN1, CIN2, CIN3, and invasive cervical carcinoma (ICC), evaluated using the immunohistological score.

	n	p16^INK4A ^immunostaining*							
		
		0	2	3	4	5	6	7	8
Normal	10	7 (70.0)	3 (30.0)	0 (0)	0 (0)	0 (0)	0 (0)	0 (0)	0 (0)
CIN1	249	47 (18.9)	22 (8.8)	18 (7.2)	35 (14.1)	19 (7.6)	20 (8.0)	12 (4.8)	76 (30.5)
CIN2	233	10 (4,3)	11 (4.7)	14 (6.0)	15 (6.4)	13 (5.6)	18 (7.7)	7 (3.0)	145 (62.2)
CIN3	181	3 (1.7)	0 (0)	3 (1.7)	3 (1.7)	2 (1.1)	3 (1.7)	5 (2.8)	162 (89.5)
ICC	133	2 (1.5)	0 (0)	2 (1.5)	1 (0.8)	1 (0.8)	1 (0.8)	3 (2.3)	123 (92.5)

* Samples were assigned an immunohistological score (0 – 8) according to the intensity of staining and the proportion of stained cells in the cervical epithelium. The total score was the sum of score for stain intensity (0 – 3 points: 0 – no staining; 1 – weak staining; 2 points – moderate staining; and 3 – strong staining) and the score for propoertion of epithelial cells stained (0 – 5 points: 0 – no staining; 1 – <1% nuclei positive; 2 – 1% – 10% nuclei positive; 3 – 11% – 33% nuclei positive; 4 – 34% – 66% nuclei positive; and 5 – > 66% of nuclei positive).

#### 1. Simple (positive vs negative)

Overexpression of p16^INK4A ^(moderate or strong staining in more than 10% of epithelial cells) was seen in 72.3% of CIN1, 91.0% of CIN2, 98.3% of CIN3, and 98.5% of invasive cervical carcinomas (Table 1). All normal cervical epithelium was 16^INK4A ^negative.

#### 2. Semi-quantitative scoring (0 – 8 points)

The distribution of p16^INK4A ^scores in cervical specimens with different grades of cervical neoplasia is shown in Table 2. Normal cervical epithelium showed consistently low immunohistological scores from 0 – 2. Specimens with dysplastic cervical epithelium showed high immunohistological scores for p16^INK4A^, and these increased with increasing CIN grade. The semi-quantitative scoring system for p16^INK4A ^expression was easy to perform, and it gave a more detailed picture of the variable positive staining seen in neoplastic lesions. However, a small but definite minority of specimens with lower grades of dysplasia had low or negative p16^INK4A ^scores (0 – 2), comprising some 9% of CIN1 and 5% of CIN2 lesions. Thus, also this immunohistological score did not allow an absolute cut-off point for p16^INK4A ^expression in relation to dysplasia or cancer to be identified, and the information obtained using the more complex protocol was in principle no different to that obtainable with the more simple positive/negative system of evaluation.

Using non-parametric statistic tests for trend across ordered groups statistical significant differences (ρ ≤ 0.01) between groups (normal tissue, CIN1, CIN2, CIN3 and ICC) were found using both evaluation methods.

## Discussion

We report the largest immunohistological study to date to look at the association between p16^INK4a ^expression and cervical neoplasia. Immunohistological expression of p16^INK4a ^was seen only in dysplastic/neoplastic cells, and was never observed in normal cervical epithelium. Thus, p16^INK4a ^expression appears to be a robust, specific and sensitive biomarker of cervical neoplasia, confirming the results of previous smaller series [17-20]. Although other pathways cannot be ruled out, increased expression of p16^INK4a ^in the setting of CIN probably occurs mainly as a result of inactivation of RB by high-risk HPVs. Circumstantial support for this premise comes from the observation that increasingly high p16^INK4a ^expression scores were seen in cervical specimens showing higher grades of CIN or invasive carcinoma, lesions known to be closely associated with high-risk HPV infection.

Most cases of CIN1, and a large proportion of cases of CIN2 and CIN3 can be expected to regress spontaneously [26]. In many other cases, the grade of dysplasia will show stable persistence. Thus, only a few percent of women with CIN1 and CIN2 lesions, and only a slightly larger minority of women with CIN3 will progress to develop invasive cancer if left untreated. This emphasizes the need for predictive biomarkers that can identify those women with cervical dysplasia who may be at risk of developing higher grades of CIN or carcinoma. However, the picture is further complicated by the fact that a large proportion of women diagnosed with cervical carcinoma have not previously had a preinvasive cervical lesion diagnosed. In our database, only 9% of women with cervical carcinoma had been previously registered with preinvasive cervical lesions. This limits the ability to study the role of p16^INK4a ^expression as a predictive marker for the development of invasive cancer, and even in the large series of cases reported here we did not have enough cases with multiple cervical biopsies to perform an analysis of p16^INK4a ^expression in specimens from individual patients over time.

Whilst the association between increasing levels of p16^INK4a ^expression and higher grades of cervical dysplasia was striking in our material, a small minority of cases with CIN was negative for p16^INK4a^. Naturally, it would be of value to know whether cases such as these would have regressed spontaneously – i.e. whether p16^INK4a ^expression is a predictive marker for progression in the cervix. Unfortunately, this cannot be determined from our data. Firstly, long-term patient follow up is not yet available on our study cohort. Secondly, most of our study specimens are removed as a part of the CIN treatment, biopsy being followed by therapeutic ablation of the remaining dysplastic epithelium, thus changing the natural history of the lesion.

The key differences between our study and similar previously reported IHC studies are firstly, the number of cases analysed and secondly, the inclusion of large numbers of premalignant intraepithelial cervical lesions. One advantage of the TMA technique is that all samples are treated with an identical staining protocol during analysis. Clearly however, this uniformity at the staining stage cannot compensate for analytical artefacts caused by eventual differences in the tissue fixation (e.g. fixation type or duration) of the original donor tissues. This is also a potential source of error when assessing whole tissue sections, although in individual cases this can be allowed for (if suspected) by subjecting parallel whole sections to different antigen retrieval and staining protocols. In practice however, this is difficult to apply to series of cases, and would be logistically impossible in a study of 796 tissues in whole sections. Fortunately, the high-throughput nature of TMA technology can to a large extent allow for errors such as those associated with differences in tissue fixation and preparation, simply because these errors are "diluted" by the size of the study cohort. TMAs can contain samples from hundreds of different donor specimens, all of which can be stained for a particular marker in a single experiment. In addition to allowing efficient high-throughput molecular profiling of large case-series, the TMA technique has other advantages. All tissue samples within a TMA are analysed simultaneously using identical reagents. By reducing the number of sections examined, assay conditions can be more easily standardised, reducing artefactual variation in staining and improves the quality of data that can be obtained. Furthermore, since only a small part of each donor tissue block is used in constructing the TMA, the technique helps to conserve scarce archival tissues for future studies. Our study provides further evidence of the value of using TMAs when performing gene expression analyses in large specimen cohorts in the search for novel diagnostic, prognostic and predictive biomarkers.

The main potential disadvantage to using the TMA technique lies in the risk that the small sample used for constructing the TMA may not be representative of the whole tissue section from the original specimen. This risk is increased if the target molecule being analysed is only rarely found within the study tissue, or shows an uneven distribution in the tissue. Prior to our main study, we carried out a pilot study comparing the effect of using multiple cores and cores of different calibre on assay results compared with whole sections (data not shown). We established that 1 mm tissue cores from target lesions were most appropriate for constructing our TMAs, compared with 0.6 mm and 2 mm cores. This was a suitable compromise, 1 mm cores being large enough to minimise problems of poor representativity (compared with whole sections) but small enough to allow high-density TMAs to be constructed.

There is no agreement on the optimal protocol for evaluating p16^INK4A ^expression in the diagnostic setting. We compared the use of a simple positive *vs. *negative score with a more complex, but still easily applied semi-quantitative immunohistological scoring system [25]. The latter gave a more detailed picture of the spread of expression scores and can be recommended for studies. However, the two systems gave similar overall results, and the simpler score appears to be quite adequate in a diagnostic setting.

Thus HPV positive tumours are characterized by high expression of p16^INK4A ^[19,27-30]. Moreover, since transcription of the E7 oncogene is required for p16^INK4A ^upregulation, it has been suggested that carcinomas overexpressing p16^INK4A ^represent those tumours in which HPV has been involved in the carcinogenic process [31].

## Conclusion

We concluded that in large numbers of sections we were able to prove that immunohistochemical detection p16^INK4a ^expression can be used as a specific diagnostic marker of all degrees of cervical dysplasia and cervical cancer, and possibly as a surrogate marker for HPV infection, due to the relationship between p16^INK4A ^and HPV E7 inactivated RB protein. The simple scoring system of a positive *vs. *a negative score seems to be adequate for practical diagnostic purposes.

## Abbreviations

CIN: Cervical intraepithelial neoplasia; HPV: Human papilloma virus; TMA: Tissue microarray.

## Competing interests

The authors declare that they have no competing interests.

## Authors' contributions

IL obtained the diagnostic material from the archive of Pathological Department, carried out immunohistochemic tests and drafts the manuscript. ML carried out pathological examination. SHD carried out pathological examination, participated in the coordination of the study and contributed to the preparation of manuscript. JK participated in the coordination of the study, and the final preparation of manuscript. All authors read and approved the final manuscript.
